# The Janus-faced role of TRPM2-S in retroperitoneal liposarcoma via increasing ROS levels

**DOI:** 10.1186/s12964-022-00873-9

**Published:** 2022-08-25

**Authors:** Xiangji Li, Fanqin Bu, Shixiang Ma, Ferdinando Cananzi, Yu Zhao, Mengmeng Xiao, Li Min, Chenghua Luo

**Affiliations:** 1 Department of Retroperitoneal Tumor Surgery, Peking University International Hospital, Peking University Eighth School of Clinical Medicine, Beijing, 102206 People’s Republic of China; 2 Department of Gastroenterology, Beijing Friendship Hospital, Capital Medical University, National Clinical Research Center for Digestive Disease, Beijing Digestive Disease Center, Beijing Key Laboratory for Precancerous Lesion of Digestive Disease, Beijing, 100050 People’s Republic of China; 3 Department of Biomedical Sciences, Humanitas University, 20089 Milan, Italy

**Keywords:** TRPM2-S, Retroperitoneal liposarcoma, ROS, Dual effects, Prognosis

## Abstract

**Background:**

Retroperitoneal liposarcoma (RPLS) is a specific soft tissue sarcoma with a high recurrence rate. The short isoform of transient receptor potential cation channel subfamily M member 2 (TRPM2-S) plays an important role in the regulation of reactive oxygen species (ROS). However, the association between TRPM2-S and RPLS and its underlying mechanisms remains unclear.

**Methods:**

The expression of both TRPM2-S and TRPM2-L in RPLS tissues was verified by kimmunohistochemistry (IHC). The regulation on Ca^2+^ influx by TRPM2-S was evaluated by Fluo-4 AM staining. The effect of TRPM2-S on cell proliferation and apoptosis was tested by 5-Ethynyl-2′-deoxyuridine (EdU) staining and Flow cytometry respectively. The level of cellular ROS was assessed by the DCFH-DA probe. Different concentrations of H_2_O_2_ were used to provide oxidative stress on RPLS cells. The underlying mechanisms were further explored by Western blotting.

**Results:**

The IHC assays showed that TRPM2-S, but not TRPM2-L, was prognostic in RPLS. Low TRPM2-S level was associated with poor disease-free survival (DFS). Calcium influx signal intensity was significantly decreased under TRPM2-S overexpression, which resulted in a decrease in the levels of FOXO3a and PTEN. Correspondingly, the levels of pERK, pAKT, pP65, pGSK-3β, Bcl-2, and β-catenin were upregulated, and cellular ROS was gently increased under TRPM2-S overexpression. Moreover, TRPM2-S slightly promoted cell proliferation and inhibited apoptosis of RPLS cell lines under normoxia, but largely increased apoptosis rates under oxidative stress. The cleaved caspase3 was significantly upregulated by TRPM2-S overexpression under oxidative stress. N-Acetyl-l-cysteine (NAC), a small molecule antioxidant, could largely rescue RPLS cells from the apoptosis induced by H_2_O_2_.

**Conclusion:**

TRPM2-S exerts Janus-faced effects in RPLS by increasing the ROS levels via inhibition on FOXO3a, which promotes cell proliferation under normoxia but induces apoptosis under oxidative stress.

**Video abstract**

**Supplementary Information:**

The online version contains supplementary material available at 10.1186/s12964-022-00873-9.

## Introduction

Retroperitoneal liposarcoma (RPLS) is a kind of soft tissue sarcoma with an incidence of 0.2–0.5 per 100,000 residents [1]. Recently, complete surgical resection (R0) remains the most effective treatment recommended for RPLS patients when feasible [2–7]. However, R0 surgical excision is challenging, rendering RPLS an uncontrollable local recurrence rate (LR) [8, 9]. Moreover, the application of both chemotherapy and radiotherapy are under dispute for the treatment of RPLS, and no well-established drug targets have been identified owing to the very limited understanding of the molecular mechanisms of RPLS.

The transient receptor potential melastatin 2 (TRPM2) is a monovalent and divalent cation-permeable ion (including K^+^, Na^+^, and Ca^2+^) channel with six transmembrane domains [10]. Numerous studies reported that TRPM2 was widely expressed in various tumor cells including neuroblastoma [11], gastric cancer [12], pancreatic cancer [13], acute myeloid leukemia [14], and prostate cancer [15]. TRPM2 accelerated tumor cell proliferation and maintain their survival via multifarious regulation mechanisms, including activating JNK-signaling, motivating PKC/MAPK pathways, and enhancing mitochondrial function by promoting mitophagy and suppressing reactive oxygen species (ROS) levels. Hitherto, at least four splice variants of TRPM2 have been reported [16–18]. The short isoform (TRPM2-S) without the four C-terminal transmembrane domains and the putative Ca^2+^ pore has been proved to repress the calcium influx through binding to TRPM2-L [18]. However, little is known about the role of TRPM2-S in malignancy, especially in RPLS.

We firstly identified TRPM2 as a key prognostic gene in RPLS from the TCGA-SARC dataset. We found that RPLS patients with higher TRPM2 expression levels showed better DFS. However, the RNA-seq data could not well distinguish the exact expression of different splice variants. Therefore, here we further determined that TRPM2-S was the “real” prognostic biomarker in RPLS by IHC staining with antibodies targeted on different sites of TRPM2. We also constructed RPLS cell lines (94T778 and SW872) stably expressing TRPM2-S to reveal the function of TRPM2-S splice variant and its bipolar function in RPLS via increasing ROS levels.

## Materials and methods

### Patients and tissue specimens

Specimens of RPLS tissues were obtained from patients who underwent surgery during 2016–2019 at the Peking University International Hospital, Peking University Health Science Center. The detailed clinicopathological characteristics of the 98 RPLS patients for immunohistochemical analysis are shown in Table 1 and Additional file 1: Table S1 and Additional file 2: Table S2. The experiments were undertaken with the understanding and written consent of each subject. The study protocol conformed to the standards set by the Declaration of Helsinki and was approved by the Ethics Committee of Peking University International Hospital, Peking University Health Science Center (WA2020RW29).

**Table 1:** Clinicopathological characteristics of the 98 RPLS patients for IHC

Clinicopathological characteristics	N (%)
*Gender*	
Male	51 (52)
Female	47 (48)
*Age (years)*	
≤ 55	48 (49)
> 55	50 (51)
*Tumor number*	
Single	28 (28.6)
Multiple	66 (67.3)
Unknown	4 (4.1)
*Tumor size (cm)*	
< 10	23 (23.5)
10–20	36 (36.7)
≥ 20	35 (35.7)
Unknown	4 (4.1)
*Histological subtype*	
Well-differentiated	29 (29.6)
Dedifferentiated	53 (54.1)
Myxoid/round cell	6 (6.1)
Pleomophic	7 (7.1)
Unknown	3 (3.1)

### Immunohistochemistry (IHC)

The TRPM2-L and TRPM2-S & -L antibodies for immunohistochemistry were purchased from Abcam (Cat No: ab11168) and Bioss (Cat No: bs-2888R), respectively. With deparaffinization for 15 min × 3 in dimethylbenzene and routine hydration, the tissues were soaked in phosphate buffer saline (PBS) for 10 min and then performed high-pressure antigen retrieval (Tris–EDTA PH = 9.0) for 2.5 min. After being treated with 3% endogenous catalase blocker (ZSBIO, PV-6000) for 10 min, the tissues were incubated in goat serum (ZSBIO, ZLI-9022) for the blocking of nonspecific reaction and then incubated with primary antibody (TRPM2-L = 1:500–1000; TRPM2-L and TRPM2-S = 1:500–2000) at 4 °C overnight. On the next day, tissues were washed routinely and incubated with goat anti-rabbit secondary antibody (ZSBIO, PV-9000) for 1 h at room temperature, then washed and stained with DAB reagents (ZSBIO, ZLI-9018). Then hematoxylin staining, 1% hydrochloric acid alcohol differentiation, ammonia water anti-blue and neutral gum sealing.

The staining extent was scored as follows: 0, negative; 1, 1–33%; 2, 34–66%; 67–100%. The intensity score was defined as negative, low-expression, medium-expression, and high-expression, which were documented as 0, 1, 2, and 3 respectively. The correlation between TRPM2-L and TRPM2-L & TRPM2-S expression and disease-free survival of RPLS patients was evaluated. The IHC results were evaluated by pathologists, and the final scores were determined by multiplying the intensity score by the extent score.

### Cell culture and transfection

The human retroperitoneal liposarcoma cell lines SW872 and 94T778 (separately purchased from Shanghai Institute of Cell Biology, Shanghai, China) were used in this study. Cells were separately cultured in 90% PRIM-1640 (CORNING) or 90% Leibovitz’s L-15 (CORNING) and 10% fetal bovine serum (Gibco), with a temperature at 37 °C in a humidified atmosphere of 5% CO_2_. Cell transfection was performed using 5 ul Lipofectamine 3000 (Invtrogen, lot: 2004186) and 1.5 ug plasmids, 4 ul P3000 according to the manufacturer’s protocol. The transfection efficiency was confirmed by western blot.

### Plasmids and antibodies

pcDNA3.1-TRPM2-S-Flag was synthesized by Youbio Co., Ltd. The following antibodies were used in this study: anti-TRPM2-L rabbit polyclonal antibody (ab11168; Abcam); anti-TRPM2-S & -L rabbit polyclonal antibody (bs-2888R; Bioss); anti-Flag (K200001M; Solarvbio); anti-PTEN (60300-1-Ig; Proteintech); anti-pAKT (66444-1-Ig; Proteintech); anti-pGSK3β (#9327S; Cell signaling technology); anti-β-Caternin (#8480S; Cell signaling technology); anti-NF-Kβ (#3033S; Cell signaling technology); anti-pERK (#4370S; Cell signaling technology); anti-Bcl-2 (#3498S; Cell signaling technology); anti-cleaved caspase3 (#9664S; Cell signaling technology); anti-GAPDH mouse monoclonal antibody (60004-1-Ig; Proteintech).

### Measurement of intracellular ROS levels

The intracellular ROS levels were measured using a Reactive Oxygen Species Assay Kit (Beyotime Biotechnology, China); 2′,7′-dichlorofluorescein-diacetate (DCFH-DA), which is easily oxidized to fluorescent dichlorofluorescein (DCF) by intracellular ROS, is its principal component, and therefore, the ROS levels were quantified. Briefly, the cells were seeded in 6-well plates and transfected with TRPM2-S plasmid. After 48 h later, cells were washed with PBS 3 times and harvested with trypsinization. Then centrifugation and resuspension, and subsequently incubated with DCFH-DA for 20 min at 37 °C. Finally, the cells were re-seeded in 96-well plates (4 × 10^4^ per well and measured at 488 nm excitation and 525 nm emission by a fluorescence spectrophotometer (SpectraMas, USA).

### Cytosolic-free calcium measurement

Fluo-4 AM (Beyotime Biotechnology, China) was used to measure intracellular calcium according to the manufacturer’s guideline. Simply, cells transfected with pcDNA3.1-TRPM2-S-Flag and empty vector were seeded in 6-well plates. After 48 h later, cells were washed with PBS 3 times and incubated with Fluo-4 AM working solution for 40 min. Then, cells were washed with PBS three times and harvested with trypsinization. After centrifugation and resuspension, cells were re-seeded in 96-well plates and analyzed the intracellular calcium with fluorescence spectrophotometer (SpectraMas, USA).

### EdU cell imaging

1 × 10^4^ cells were seeded in 24-well culture plates 36 h after transfection. After 12 h of culture, 50 uM EdU (Cell Light™ EdU Apollp®643 In Vitro Imaging Kit, Lot: C10310-2) was added and incubated at 37 °C for 2 h. Excess EdU was eluted with PBS, then after fixation, glycine treatment, membrane permeability, cells were incubated with 300 ul of Apollo staining reaction solution for 30 min with gently shaking in the darkness. After washing with 0.5% Triton-X-100 and methanol enhanced bleaching treatment, the cell nucleus was stained with 1 × Hoechst 33342 reaction solution for 30 min with gently shaking in the darkness. Cells fluorescence was observed using a fluorescence microscope (OLYMPUS DP72) and Apollo/Hoechst positive ratio was calculated.

### Live-cell imaging

1.5 × 10^3^ cells were seeded in 96-well culture plates 48 h after transfection. Cell proliferation was monitored for at least 50 h by a long-term process live-cell analysis system IncuCyte S3 (Essen Instruments, Ann Arbor, MI, United States). Photographs of cells were taken at 6 h intervals from four separate regions per well with 10 objectives. Values from four regions of each well were pooled and averaged across six replicates.

### Western blotting

Proteins for conventional western blotting were obtained by lysing cells in RIPA lysis buffer (50 mM Tris/HCl, 150 mM NaCl, 1% NP-40, 0.5% sodium deoxycholate, and 0.1% SDS) and disruption with gentle sonication (on 3 s/off 3 s for 10 cycles). The protein concentration was confirmed by a bicinchoninic acid Protein Assay Kit (Thermo Fisher, Waltham, MA, USA) and was normalized with 5 × loading buffer and ddH_2_O. Denatured total protein was separated by 10% and 12% SDS/PAGE and then transferred to nitrocellulose membranes. Nonspecific binding sites were blocked for 2 h at room temperature using 5 and 8% (w/v) milk (skim milk powder in TBST). The proteins were incubated overnight at 4 °C with the primary antibody. After washing three times with TBST, the blot was incubated with the HRP-conjugated secondary antibody for 1 h at room temperature and visualized with an enhance chemiluminescence system (Bio-Rad, Hercules, CA, USA).

### Flow cytometry for apoptosis (Annexin-V & 7-AAD)

Cells were washed with PBS after transfection for 48 h (overexpression group and negative control group with or without 200 umol/L H_2_O_2_ and N-Acetyl-l-cysteine (NAC) treatment for 24 h to induce or anti-apoptosis) and were then harvested using 0.25% trypsin (all supernatant liquid was collected). After centrifugation (1500 rpm) for 15 min, the supernatant liquid was discarded, and the precipitation was resuspended in 1 mL of binding buffer for another centrifugation. Then, the cells were resuspended in 250 ul of binding buffer, and 5 ul each of annexin V-FITC and 7-AAD-Percp-Cy5.5 (BD Biosciences, San Jose, CA, USA) were added. The cells were analyzed by a FACSVerse (Becton–Dickinson, FranklinLakes, NJ, USA) after incubation in the dark for 15 min.

### Propidium iodide (PI) staining for apoptosis

2 × 10^4^ cells were seeded in 24-well culture plates 36 h after transfection. After 12 h of culture, H_2_O_2_ with different concentration gradients (including 0, 100, 200, and 300 umol/L) was added and incubated at 37 °C for 24 h. then cells were washed with PBS and incubated with 300 ul of Propidium iodide (PI) staining reaction solution for 20 min in darkness. After washing the excess PI, cells fluorescence was observed using fluorescence microscope (OLYMPUS DP72) and PI/Cell counts positive ratio was calculated.

### Statistical analysis

SPSS 28.0 (IBM, Armonk, NY, USA) and Prism 9 for Mac OS were used for statistical analysis and visualization. Data are presented as the mean ± standard deviations (SDs). Student’s independent t-test was used for statistical comparisons between the experimental and control groups. Kaplan–Meier (K–M) plots was applied to assess and show the difference in disease-free survival (DFS) between subgroups. P values of < 0.05 were considered statistically significant.

## Results

### Low TRPM2-S expression predicted poor prognosis in RPLS

**Fig. 1:**
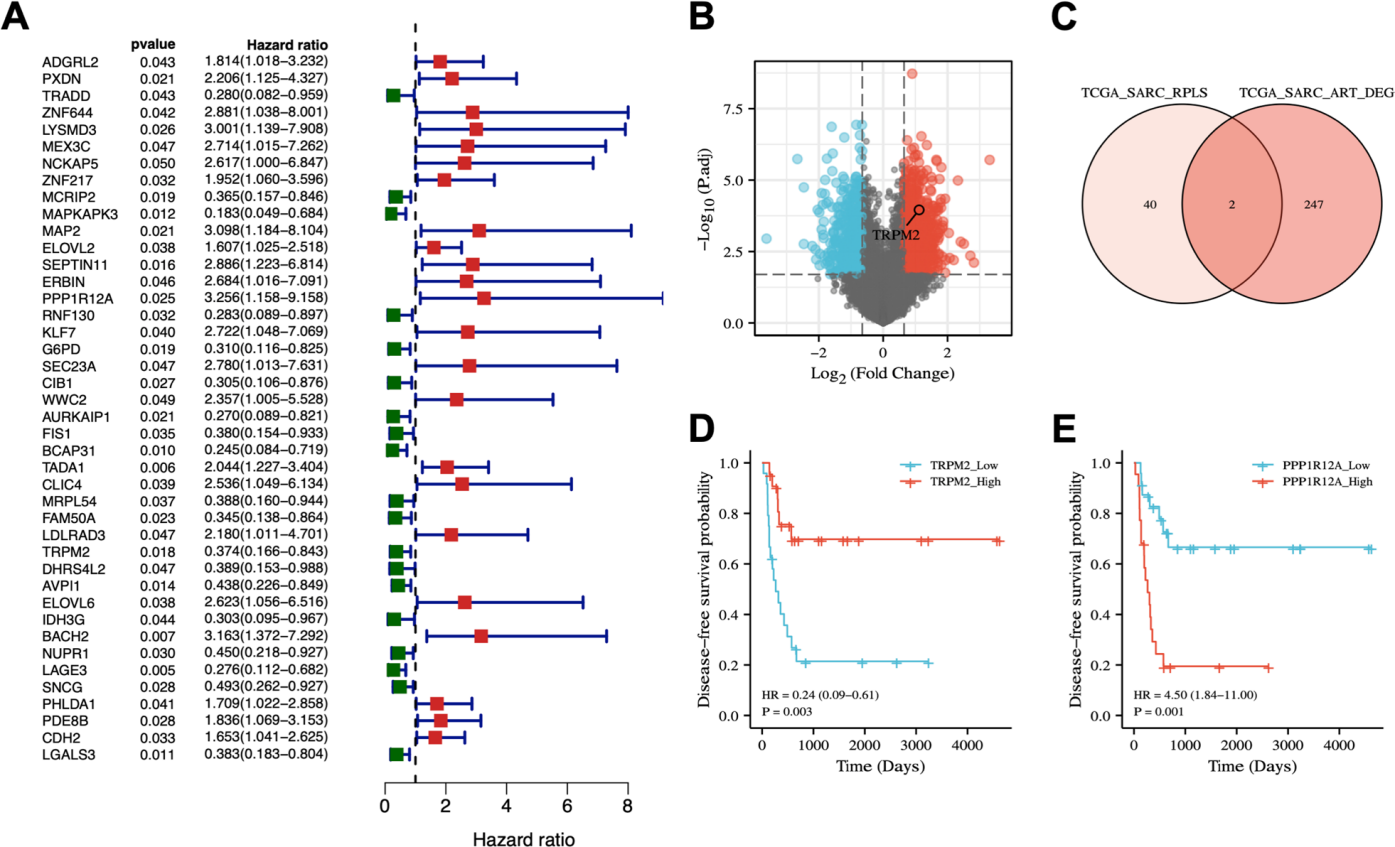
RPLS prognostic gene screening from TCGA-SARC. **a** DFS univariate Cox analysis of TCGA-SARC-RPLS and forest map showed all prognostic genes with P < 0.05. **b** The volcanic map of TCGA-SARC-ART differential gene analysis, red represents up-regulation and blue represents down-regulation. **c** Venn of TCGA-SARC-RPLS prognostic gene set (P < 0.05) and TCGA-SARC-ART differential gene set (FDR < 0.01). **d** Kaplan–Meier analysis of TRPM2 and PPP1R12A in TCGA-SARC-ART

To identify clinically relevant biomarkers in RPLS, we performed a univariate Cox analysis of disease-free survival (DFS) on 57 RPLS samples from the TCGA-SARC. DFS was chosen for the survival time analysis since the main factor affecting the survival of RPLS patients is the postoperative recurrence rate. We revealed a total of 42 prognostic genes with a P-value < 0.05 (Fig. 1a, Additional file 3: Table S3). As previously we found that adjuvant radiotherapy had positive effects for the treatment of retroperitoneal sarcomas [19], here we further investigated the RPLS patients who underwent surgery and radiotherapy, respectively. We divided the patients into two subgroups according to whether remission was achieved by radiotherapy (Fig. 1b, Additional file 4: Table S4) to screen out crucial effector genes for radiotherapy. The prognostic genes set (P < 0.05) and differential genes set (FDR < 0.01) were subsequently subjected to intersection analysis to identify genes significant for both prognosis and radiotherapy (Fig. 1c). Two genes (TRPM2 and PPP1R12A) were finally identified. The Kaplan Meier prognostic analysis indicated TRPM2 as a prognostic protective factor and PPP1R12A as a prognostic invasive factor (Fig. 1d, e). Here we selected TRPM2 for subsequent mechanism studies.

**Fig. 2:**
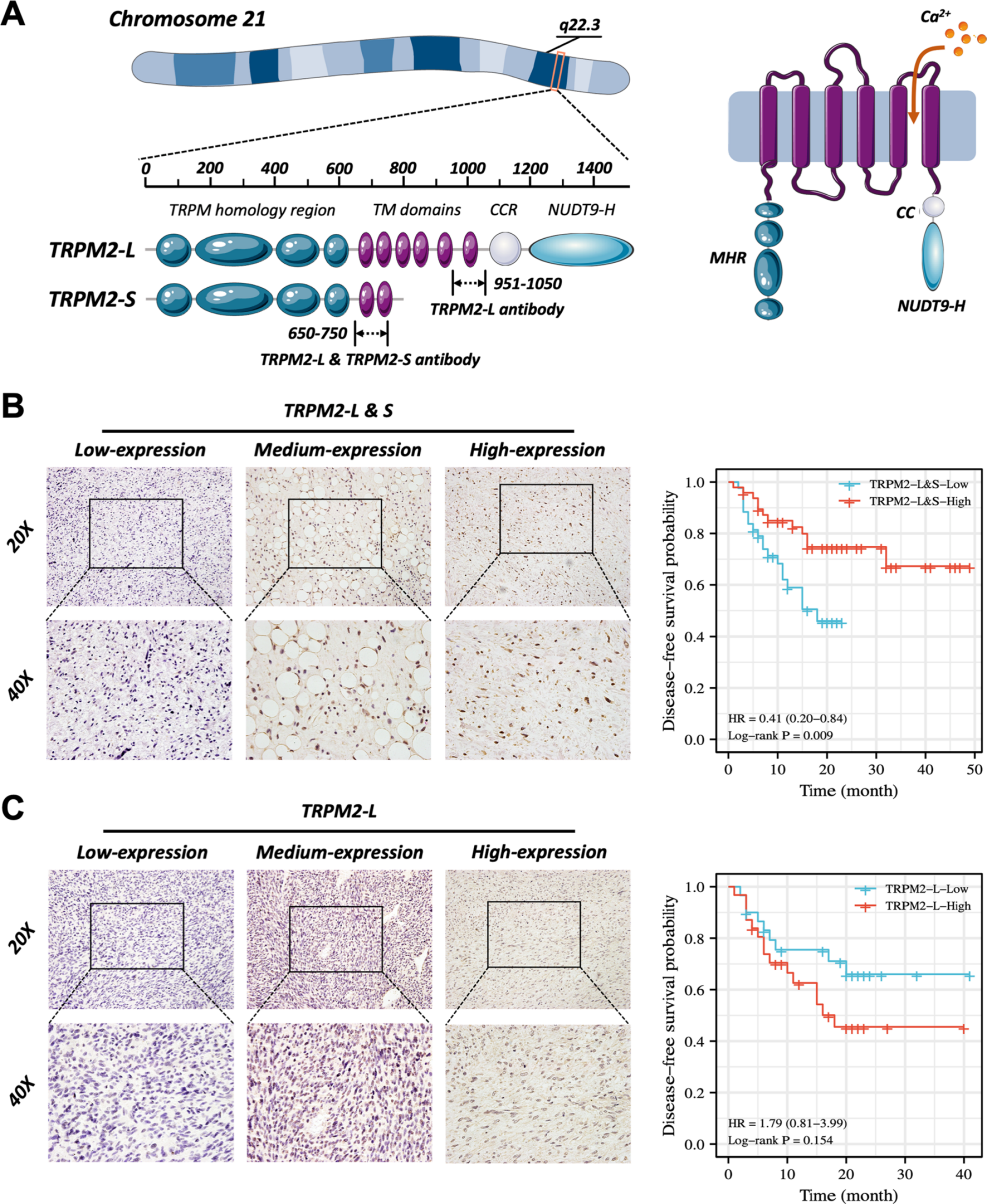
Validation of the RPLS prognostic gene TRPM2-S. **a** Schematic diagram of TRPM2 gene and encoded protein isoforms (TRPM2-L and TRPM2-S) (left panel) and membrane topology (right panel). **b** Representative RPLS staining images with different IHC scores (left panels). Log-rank analysis was performed and showed a higher DFS in the high TRPM2-L & -S expression group (right panels). **c** Representative RPLS staining images with different IHC scores (left panels). Log-rank analysis was performed and showed DFS was not significantly different between the high TRPM2-L and low TRPM2-S expression groups (right panels)

Considering TRPM2 has two different transcripts due to alternative splicing (Fig. 2a), we performed IHC staining with antibodies that recognized total TRPM2 (both TRPM2-L & TRPM2-S) and specifically recognized the TRPM2-L variant. The results indicated that patients with higher TRPM2-L & TRPM2-S had a better DFS than those with lower expression (Fig. 2b, P = 0.009), whereas there was no significant difference between patients with high- and low expression of TRPM2-L (Fig. 2c, P = 0.154). Univariate Cox regression suggested that high expression of TRPM2-L & TRPM2-S was associated with longer DFS (Table 2). Based on the above results, we concluded that TRPM2-S, but not TRPM2-L, was the “real” prognostic factor in RPLS patients.

**Table 2:** Univariate and multivariate analyses evaluating the association between covariates and DFS

Clinicopathological characteristics	Univariate analysis HR (95% CI)	P value	Multivariate analysis HR (95% CI)	P value
*Gender*				
Male	Reference			
Female	1.21 (0.60–2.42)	0.60		
*Age (years)*				
≤ 55	Reference			
> 55	1.87 (0.90–3.86)	0.09	1.30 (0.60–2.82)	0.50
*Tumor number*				
Single	Reference			
Multiple	1.37 (0.59–3.18)	0.46		
*Tumor size (cm)*				
< 10	Reference			
10–20	1.02 (0.44–2.39)	0.96		
≥ 20	0.63 (0.25–1.58)	0.32		
*Histological Subtype*				
Well-differentiated	Reference			
Dedifferentiated	3.79 (1.40–10.23)	< 0.01	3.02 (1.06–8.66)	0.04
Myxoid/round cell	–			
Pleomophic	4.00 (0.94–17.16)	0.06	3.17 (0.69–14.63)	0.14
*TRPM2-L*				
Low	Reference			
High	1.81 (0.79–4.15)	0.160		
*TRPM2-L and TRPM2-S*				
Low	Reference			
High	0.42 (0.20–0.88)	0.021	0.63 (0.29–1.37)	0.24

### ***TRPM2-S suppressed the intracellular flow of calcium ions (Ca***^***2***+^***)***

**Fig. 3:**
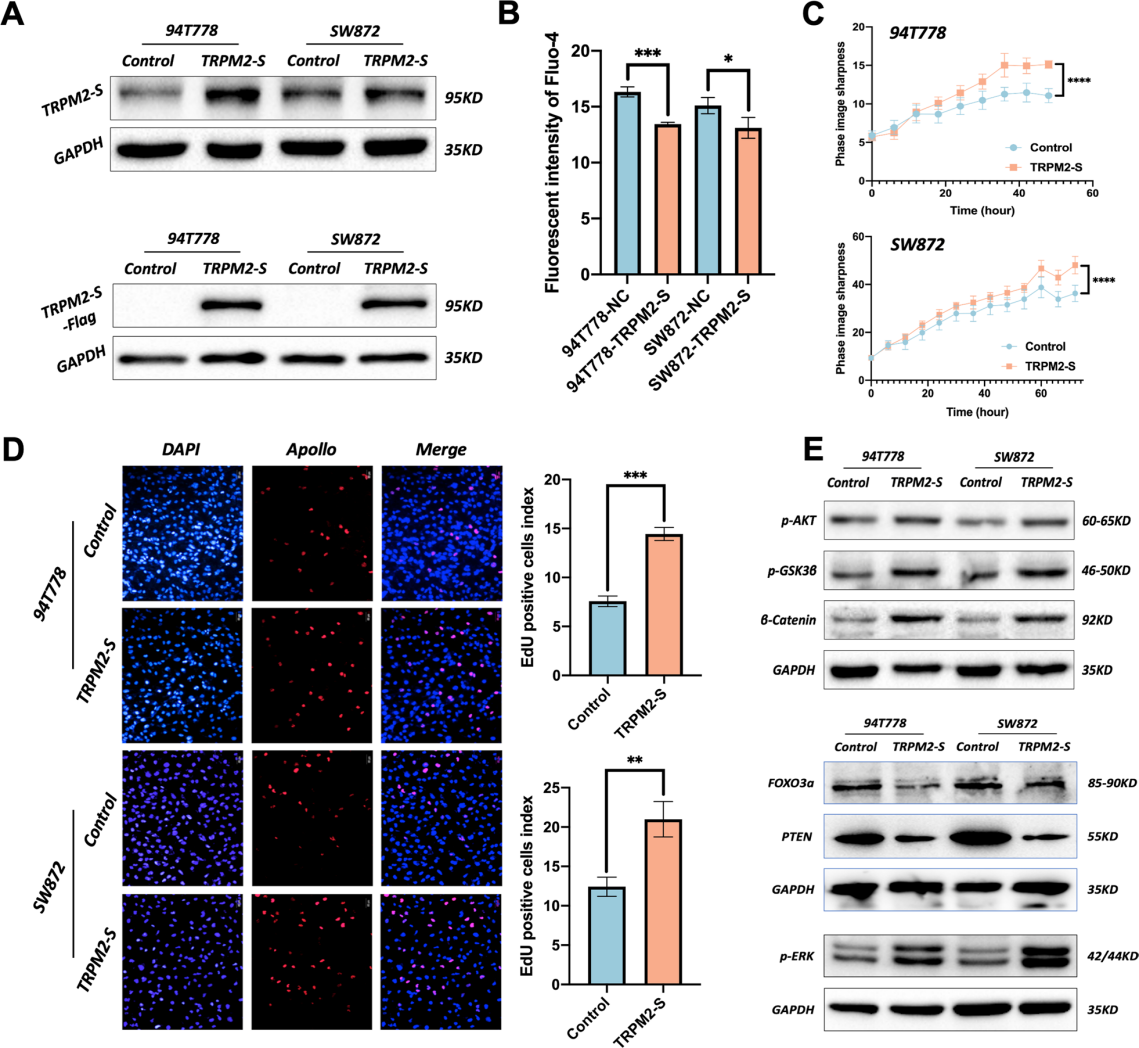
TRPM2-S promotes proliferation via activating ERK and AKT. **a** Western blotting indicated TRPM2-S expression in pcDNA3.1-TRPM2-S-Flag cells was obviously higher than in empty vector RPLS cell lines. **b** TRPM2-S inhibited the intracellular flow of Ca^2+^. **c** Growth curves of live-cell imaging showed TRPM2-S-expressing 94T778 and SW872 cells have a higher proliferative capacity than negative control cells. **d** Representative images of EdU assays (left panels). EdU showed that a significant proliferation rate was observed in TRPM2-S-expressing RPLS cell lines (right panels). **e** Western blotting revealed that p-Akt, p-GSK3β, β-Catenin, and p-ERK were upregulated, whereas FOXO3a and PTEN were downregulated in TRPM2-S-expressing cells compared with control cells

To reveal the biological function of TRPM2-S, two human RPLS cell lines (SW872 and 94T778) were transfected with pcDNA3.1-TRPM2-S-Flag, and the TRPM2-S protein expression was assessed by WB. As shown in Fig. 3a, the transfection efficiency was considered feasible. Considering that TRPM2 is a tetrameric cation channel gene, we further evaluated the function of TRPM2-S on the intracellular flow of Ca^2+^ with Fluo-4 AM reagent. The result showed that the level of cytosolic-free Ca^2+^ was significantly reduced in RPLS cells with TRPM2-S overexpression (Fig. 3b). These results demonstrated that TRPM2-S could suppress the intracellular flow of Ca^2+^.

### *TRPM2-S promoted proliferation and inhibited apoptosis *via* activating ERK and AKT*

**Fig. 4:**
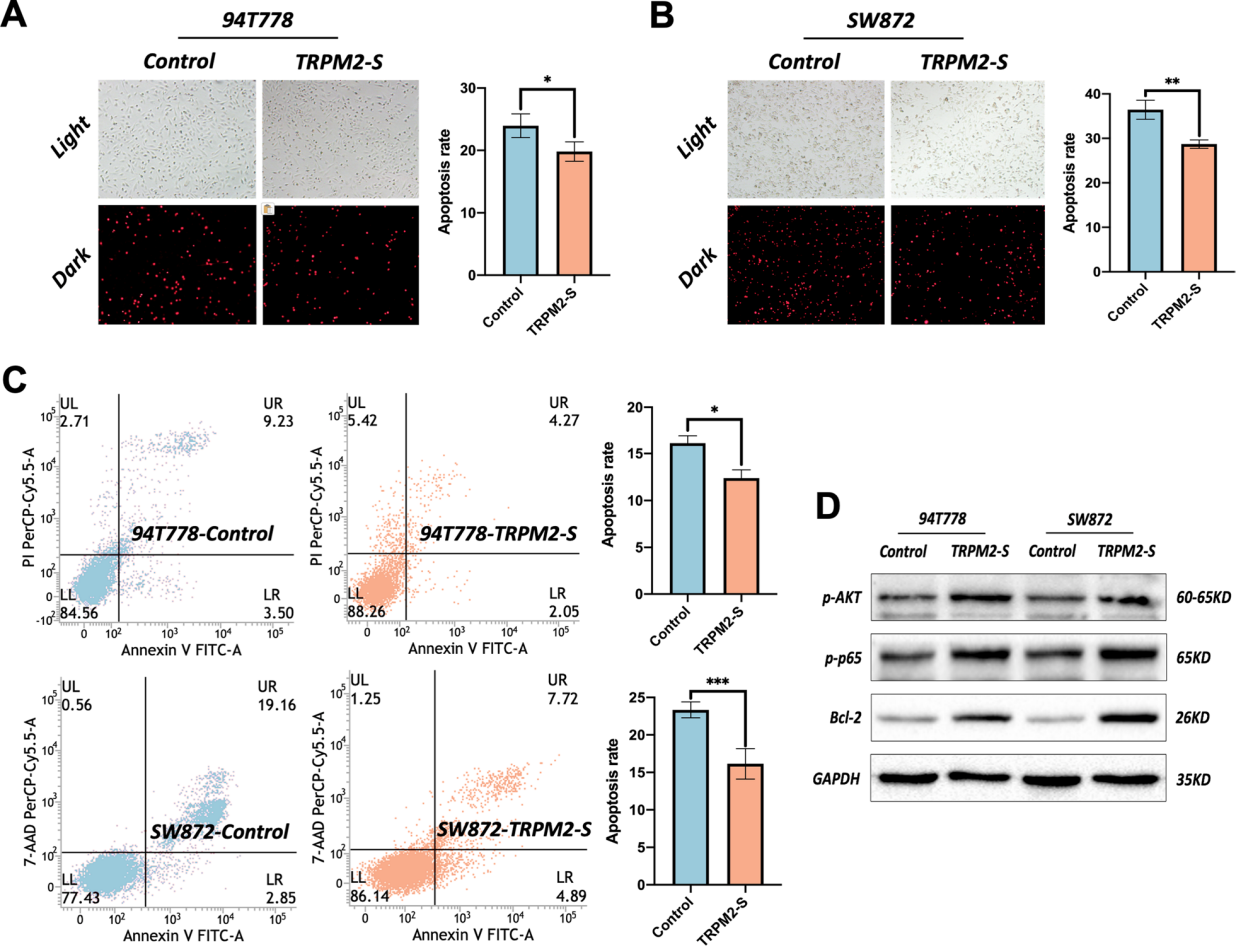
TRPM2-S inhibits apoptosis via activating AKT. **a**–**c** Representative images of PI staining (**a**, **b**) and flow cytometry for apoptosis (**c**) (left panels), both two analyses showed that apoptosis rate was obviously increased in TRPM2-S-expressing RPLS cells (right panels). **d** Western blotting revealed that p-Akt, pP65, and Bcl-2 were upregulated in TRPM2-S-expressing RPLS cells compared with control cells

**Fig. 5:**
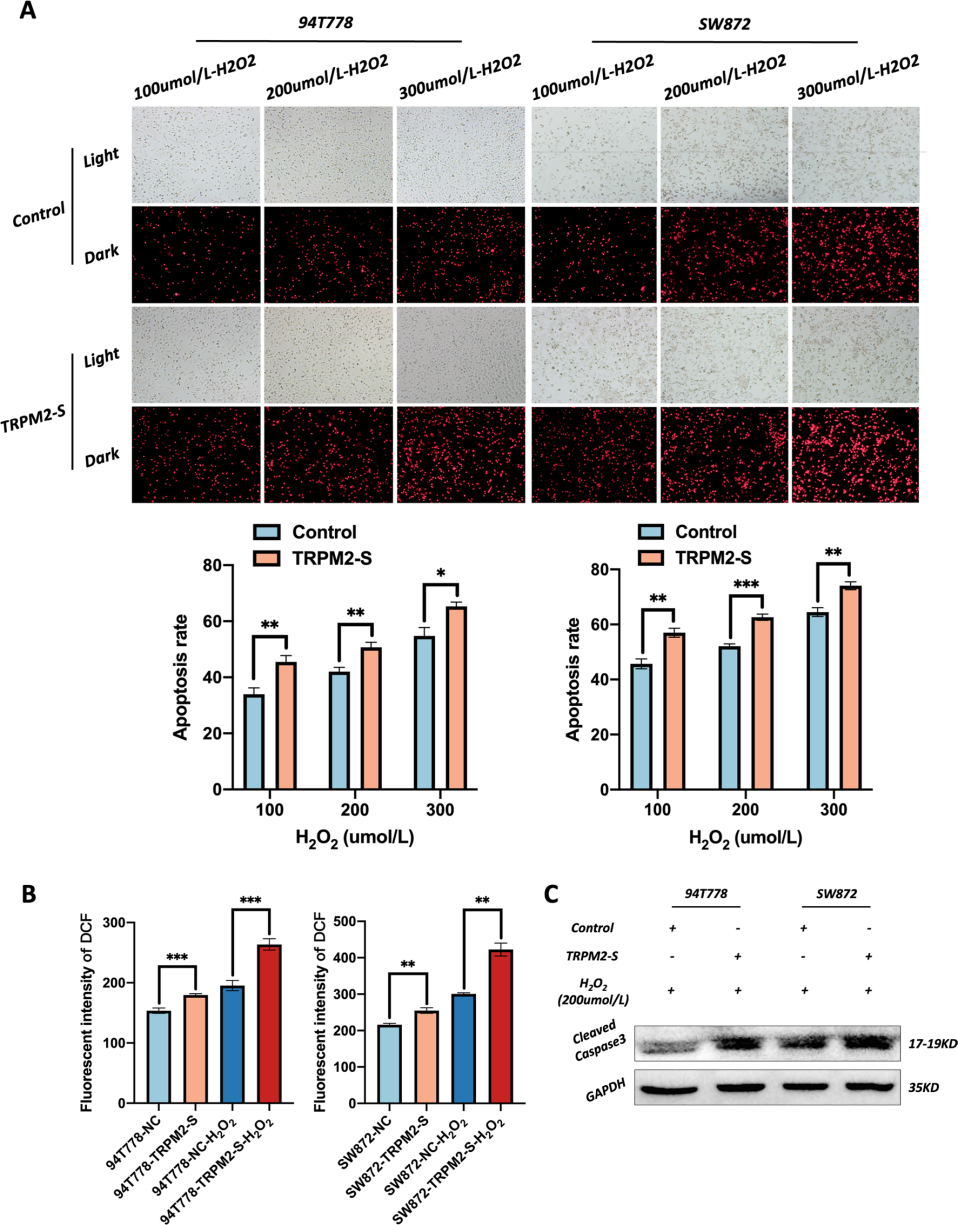
TRPM2-S increased the susceptibility to apoptosis of RPLS cells under oxidative stress. **a** Representative images of PI staining (upper panels), the analysis indicated that the apoptosis rate was significantly higher in TRPM2-H_2_O_2_ cells than control-H_2_O_2_ cells and had a concentration-dependence property (down panels). **b** ROS kit assays showed that ROS was gently increased in TRPM2-S-expressing cells but was sharply elevated in TRPM2-H_2_O_2_ cells compared with control cells and control-H_2_O_2_ cells respectively. **c** Western blotting revealed that cleaved caspase 3 was upregulated in TRPM2-S-H_2_O_2_ RPLS cells compared with control-H_2_O_2_ cells

To investigate the role of TRPM2-S, EdU, live-cell imaging, and flow cytometry (Annexin-V & 7-AAD staining) were used to evaluate the proliferation and apoptosis of 94T778 and SW872 cells. The EdU and live-cell imaging showed that cells with TRPM2-S overexpression had a higher proliferation rate compared to control cells (Fig. 3c, d). The PI staining and flow cytometry analysis indicated that TRPM2-S overexpression largely decreased the apoptosis rate of RPLS cells (Fig. 4a–c). Moreover, the phosphorylation of both ERK and AKT was significantly upregulated in cells with TRPM2-S overexpression, and other associated proteins (*e.g.* pGSK3β, β-catenin, pP65, and Bcl-2) were also upregulated (Figs. 3e, 4d). As the direct downstream of TRPM2-controlled intracellular Ca^2+^ flow, the level of FOXO3a was also significantly downregulated in the cells with TRPM2-S overexpression (Fig. 3e). Considering FOXO3a could regulate both the ERK and AKT signaling via ROS, we also evaluated the ROS level with DCFH-DA, and the result demonstrated that ROS was gently increased in cells with TRPM2-S overexpression compared with control cells (Fig. 5b). Therefore, the above results implied that TRPM2-S promoted proliferation and inhibited apoptosis of RPLS cells via activating ERK and AKT pathways.

### TRPM2-S increased the susceptibility to apoptosis of RPLS cells under oxidative stress

Considering the Janus-faced role of ROS in cancer, we also assessed the effects of TRPM2-S on cells with different oxidative stress. PI staining was used to detect the apoptosis rate of RPLS cells treated with different concentrations of H_2_O_2_. The results showed that the apoptosis rate was obviously increased in cells with TRPM2 overexpression in a concentration-dependent manner (Fig. 5a) along with a boosting of ROS levels (Fig. 5b). Then, we performed WB to explore the underlying mechanisms. The results demonstrated that cleaved caspase 3 was significantly elevated under TRPM2-S overexpression in a circumstance with high levels of H_2_O_2_ (Fig. 5c). These results revealed that TRPM2-S enhanced the apoptosis of RPLS cells by upregulating the level of cleaved caspase 3 under oxidative stress.

### NAC appeased the TRPM2-S enhanced apoptosis of RPLS cells under oxidative stress

**Fig. 6:**
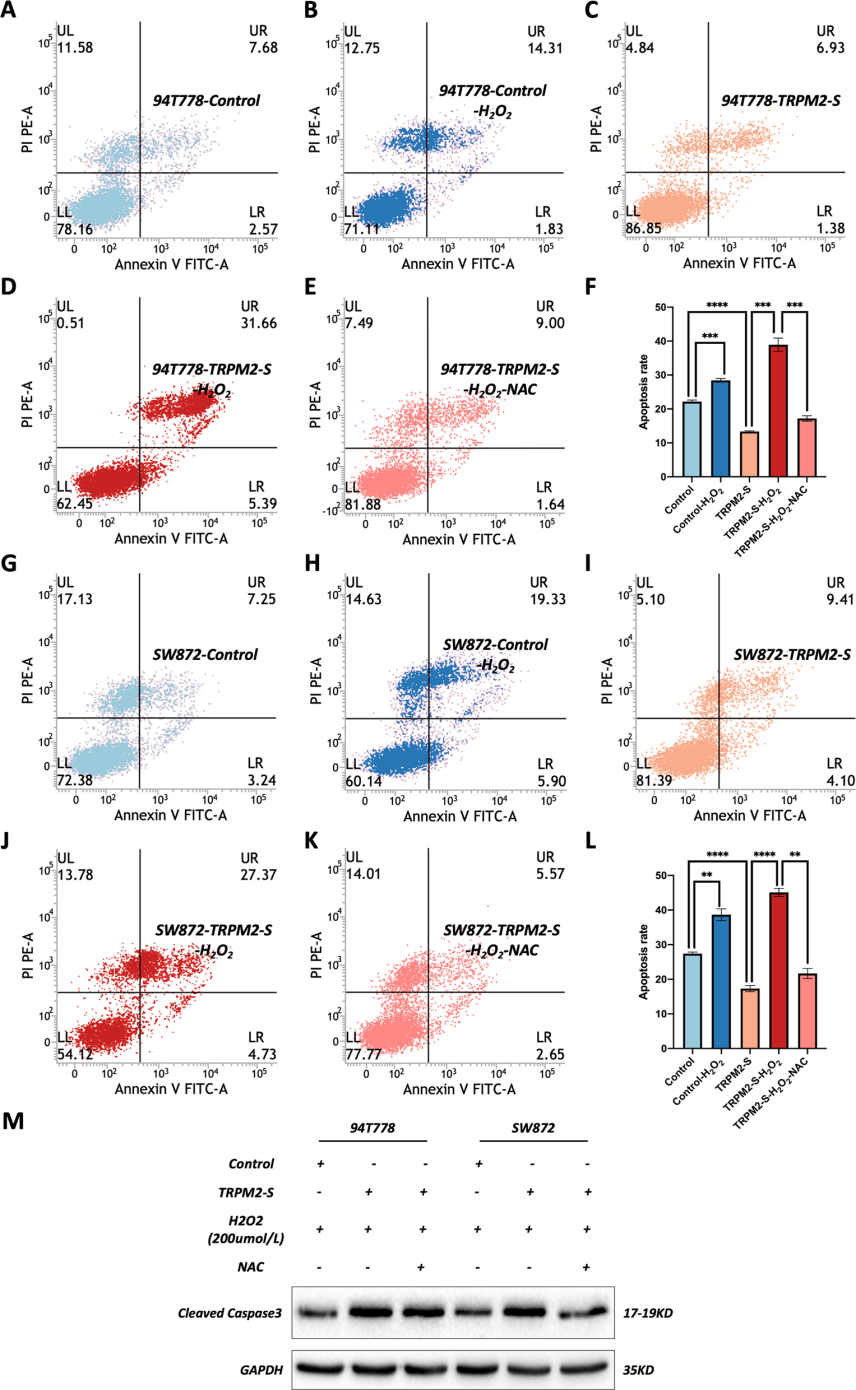
NAC appeased the TRPM2-S enhanced apoptosis of RPLS cells under oxidative stress. **a**–**e** Representative images of flow cytometry for apoptosis, including 94T778-Control (**a**), 94T778-Control-H_2_O_2_ (**b**), 94T778-TRPM2-S (**c**), 94T778-TRPM2-S-H_2_O_2_ (**d**), 94T778-TRPM2-S-H_2_O_2_-NAC (**e**). **f** Analysis of apoptosis rate of 94T778 cell. **g**–**k** Representative images of flow cytometry for apoptosis, including SW872-Control (**g**), SW872-Control-H_2_O_2_ (**h**), SW872-TRPM2-S (**i**), SW872-TRPM2-S-H_2_O_2_ (**j**), SW872-TRPM2-S-H_2_O_2_-NAC (**k**). **l** Analysis of apoptosis rate of SW872 cell. **m** Western blotting revealed that cleaved caspase 3 was upregulated in TRPM2-S-H_2_O_2_ RPLS cells but was downregulated in TRPM2-S-H_2_O_2_-NAC RPLS cells compared with control-H_2_O_2_ and TRPM2-S-H_2_O_2_ RPLS cells respectively

To further confirm that TRPM2-S enhanced apoptosis via boosting of ROS under oxidative stress, NAC, a small-molecule antioxidant, was used to attenuate the ROS levels of TRPM2-S overexpressing (After inducing oxidative stress with H_2_O_2_ for 24 h, the medium was replaced with complete medium and treated with NAC for another 24 h). We detected the apoptosis rate of RPLS cells and found that the apoptosis rate was appeased to the base level by NAC in cells with TRPM2 overexpression under oxidative stress (Fig. 6b–f, h–l). Consistent with the previous results in Fig. 4a–c, the TRPM2-S group showed a lower apoptosis level compared to the control group, while the TRPM2-S-H_2_O_2_ group showed a higher apoptosis level compared to the control-H_2_O_2_ group (Fig. 6a-d, f–j, l). Moreover, WB assays indicated that the level of cleaved caspase 3 was decreased under NAC treatment (Fig. 6m). Therefore, these results revealed that NAC could appease the TRPM2-S enhanced apoptosis of RPLS cells under oxidative stress.

## Discussion

In this report, a new isoform of TRPM2, TRPM2-S was identified and validated from the TCGA database and our REtroperitoneal SArcoma Registry (RESAR) cohort. We demonstrated that TRPM2-S inhibited calcium influx, enhanced cell proliferation, and reduced apoptosis under normoxia but induced apoptosis under oxidative stress. It is noteworthy that TRPM2-S may have a “dual role” in the determination of RPLS cell fate following whether exposure to oxidative stress.

The first major finding of this report is that RPLS patients with high-expression TRPM2-S predict a better DFS. To determine exactly which TRPM2 isoform (TRPM2-L or TRPM2-S) has a prognostic role in RPLS. IHC was performed to validate the gene in RESAR cohort, and antibodies of TRPM2-L and TRPM2-L & TRPM2-S were applied in this analysis due to the lack of antibodies to recognize TRPM2-S alone. Only patients with high expression of TRPM2-L & TRPM2-S had a better DFS, which meant TRPM2-S, not TRPM2-L, was the real prognostic gene.

To evaluate the role of TRPM2-S in cell proliferation and apoptosis, we engineered RPLS cell lines stably expressing either TRPM2-S or empty vector. Our second major finding is that TRPM2-S promotes proliferation and inhibits apoptosis of RPLS cells. It was associated with decreased FOXO3a and PTEN, gently increased ROS, increased pAkt, and pERK. Actually, trends in the alteration of these downstream proteins were consistent with the results reported by Chen et al. They demonstrated that TRPM2-S could promote neuroblastoma cell proliferation by activating ERK, Akt, and increasing the level of Glut1 [20]. Down-regulated levels of PTEN have been associated with increased cell proliferation and have been observed in large amounts of cancers [21–24]. It is generally recognized that PTEN downregulation accelerates cell proliferation by phosphorylating Akt (pAkt) [25]. In our study, we found that TRPM2-S upregulated FOXO3a-mediated ROS production in RPLS cell lines through inhibiting cation influx. ROS can inactivate PTEN via oxidation, resulting in increased pAkt [26, 27]. Besides, ROS can also increase pERK, which has been reported in many studies [28–30]. Our experiments with WB indicated the same results that decreased PTEN, increased pERK, and activated Akt and its downstream molecules (including pAkt/pGSK3β/β-Catenin and pAkt/pP65/Bcl-2). Therefore, we conclude that TRPM2-S promoted proliferation and inhibited apoptosis via activating ERK and AKT.

Different types of cancer cells have been shown to produce higher levels of ROS compared to their normal counterparts [31]. The elevated ROS is thought to be oncogenic, causing damage to DNA, proteins, and lipids, promoting genetic instability and tumorigenesis [32–34]. It also acts as signaling molecules in cancers, contributing to abnormal cell growth, metastasis, resistance to apoptosis, angiogenesis, and differentiation block [35, 36]. Our previous discussion showed that ROS plays an extremely role in the pro-proliferation and anti-apoptosis of TRPM2-S. However, it is wide awareness that toxic levels of ROS production in cancers are anti-tumorigenic resulting in an increase of oxidative stress and induction of tumor cell death [37–39]. Our third major finding is that TRPM2-S is more susceptible to low concentrations (100–300 umol/L) of H_2_O_2_ and induces RPLS cell apoptosis under oxidative stress. We consider that the susceptibility is caused by decreased FOXO3a. Subsequently, the sharply elevated ROS caused by ROS homeostatic imbalance and oxidative stimulation ultimately activates caspase 3 and triggers apoptosis. This is consistent with the previously reported findings, in which TRPM2-S decreased cell survival under oxidative stress conditions by inhibiting Ca^2+^. In those experiments, TRPM2-S expression inhibited the levels of intracellular antioxidant enzymes (including HIF1/2α, FOXO3a, and SOD1/2) as well as mitochondrial functional proteins (including NDUFA4L2, BNIP3, and Cox4.1/2) [11, 14]. However, the mechanism through TRPM2-S regulating FOXO3a here is not known. Pathways involving reduced Ca^2+^ influx through TRPM2-S may be involved in the modulation of FOXO3a expression. For example, one mechanism through which decreased Ca^2+^ influx in TRPM2-S expressing cells could reduce HIF expression by destabilizing HIF-1α. Meanwhile, decreased Ca^2+^ influx can inactivate calcineurin, which attenuates the dephosphorylation of RACK1, increasing RACK1 dimerization and reducing HIF-α levels by enhancing its ubiquitination and degradation [40]. The reduction of HIF-1/2α revoked the inhibition of Akt activation, impeding the FOXO3a level by accelerating its phosphorylation [41].

Wide excision is the main treatment for retroperitoneal liposarcoma, but the patient’s perioperative period was prolonged due to the extensive surgical ripples and massive intraoperative blood loss. In this process, ischemia/reperfusion injury and inflammation are two key factors causing oxidative stress [42]. Meanwhile, postoperative radiotherapy also provides a non-negligible pathway for the emergence of oxidative stress [43]. Based on the above inferences, we believe that surgery and radiotherapy lead to a better prognosis for patients with high TRPM2-S expression. Of course, a meticulous clinical cohort study must be designed to validate this finding, which is also the direction of our subsequent in-depth study. Besides, an obvious limitation of this study is that it does not clearly reveal the underlying mechanism of TRPM2-S-induced apoptosis under oxidative stress, but according to a series of reports by Chen et al. [11, 14, 20], we hypothesized that TRPM2-S induced apoptosis via mitochondrial pathway (endogenous apoptotic pathway) under oxidative stress.

**Fig. 7:**
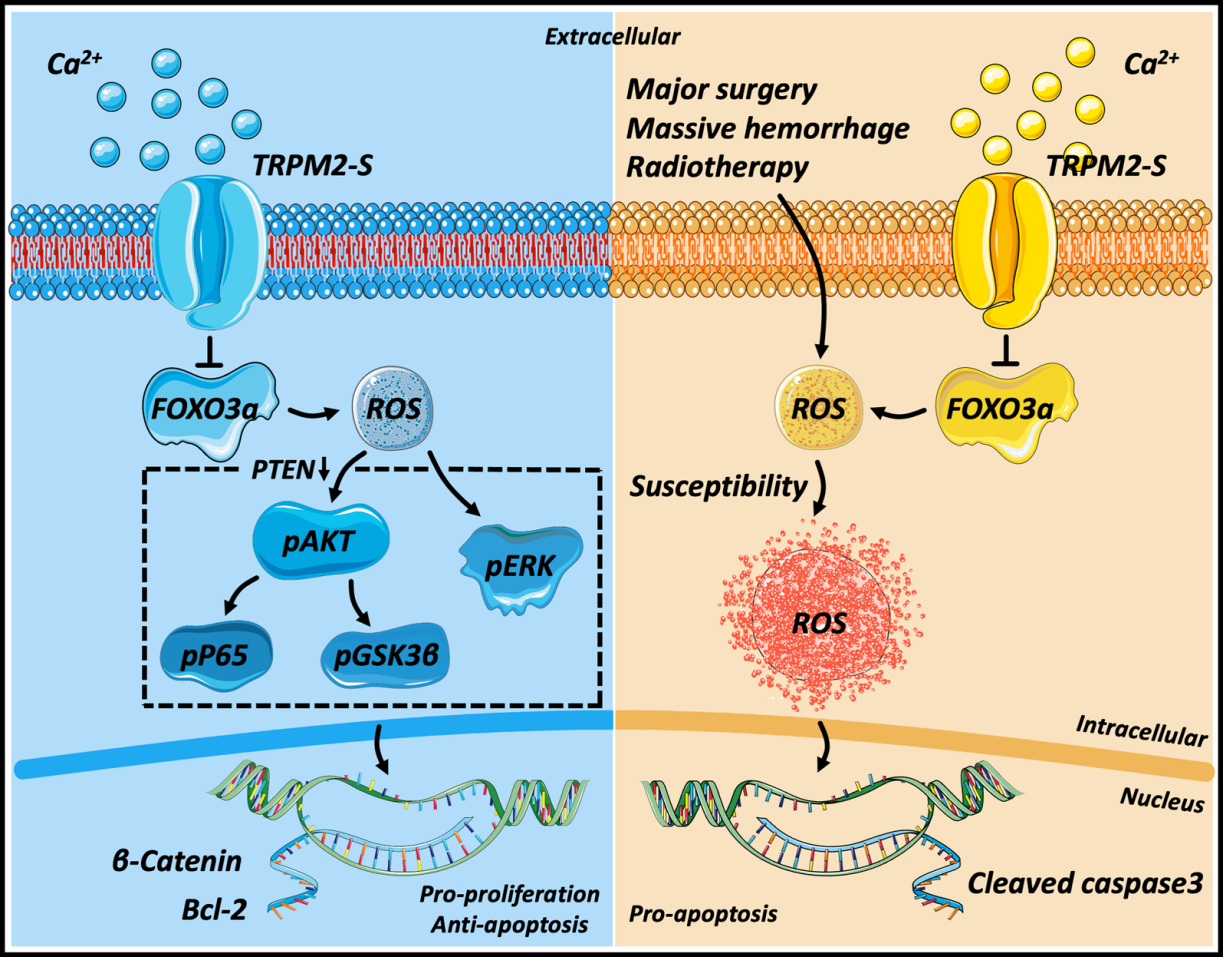
Schematic diagram of the molecular mechanism of TRPM2-S in RPLS

Taken together, TRPM2-S promotes proliferation and inhibits apoptosis via activating PTEN/Akt and ERK but induces apoptosis through reduction of FOXO3a and activation of cleaved caspase 3 under oxidative stress (The molecular mechanism is shown in Fig. 7). The pivotal point for this dual effect shift is whether the ROS concentration crosses its threshold for triggering toxicity. For this reason, therapies used to elevate ROS production may be potentially effective RPLS therapies although it is a rather challenging concept.

## Supplementary Information

**Additional file 1**. The detailed clinical characteristics and TRPM2-L-IHC score of RPLS patients.

**Additional file 2**. The detailed clinical characteristics and TRPM2-S & L-IHC score of RPLS patients.

**Additional file 3**. The DFS-prognostic genes screened by Cox analysis in TCGA-RPLS.

**Additional file 4**. The differential genes between the radiotherapy remission group and non-remission group inTCGA-SARC.

## Data Availability

The datasets and materials used for this study are available from the corresponding author on reasonable request.

## Abbreviations

RPLS: Retroperitoneal liposarcoma
TRPM2-S: The short isoform of transient receptor potential subfamily M2
ROS: Reactive oxygen species
TRPM2-L: The full-length of transient receptor potential subfamily M2
IHC: Immunohistochemistry
EdU: Ethynyl-2′-deoxyuridine
DFS: Disease-free survival
FOXO3a: Forkhead Box O3
PTEN: Phosphatase and tensin homolog
AKT: Protein kinase B
pAKT: Phosphor-AKT
pGSK3β: Phosphor-glycogen synthase kinase 3 beta
ERK: Mitogen-activated protein kinase
pERK: Phosphor-ERK
pNFKB/p65: Phosphor-nuclear factor-kappa B
NAC: N-acety1-l-cysteine
PBS: Phosphate buffer saline
DCFH-DA: 2′,7′-Dichlorofluorescein-diacetate
TCGA: The cancer genome atlas
SARC: Sarcoma
RESAR: Retroperitoneal Sarcoma Registry
